# Redox Regulation of STAT1 and STAT3 Signaling

**DOI:** 10.3390/ijms21197034

**Published:** 2020-09-24

**Authors:** Elena Butturini, Alessandra Carcereri de Prati, Sofia Mariotto

**Affiliations:** Department of Neuroscience, Biomedicine and Movement Sciences, Section of Biological Chemistry, University of Verona, Strada Le Grazie, 8, 37134 Verona, Italy; elena.butturini@univr.it (E.B.); alessandra.carcererideprati@univr.it (A.C.d.P.)

**Keywords:** STAT1, STAT3, oxidative stress, post-translational modifications

## Abstract

STAT1 and STAT3 are nuclear transcription factors that regulate genes involved in cell cycle, cell survival and immune response. The cross-talk between these signaling pathways determines how cells integrate the environmental signals received ultimately translating them in transcriptional regulation of specific sets of genes. Despite being activated downstream of common cytokine and growth factors, STAT1 and STAT3 play essentially antagonistic roles and the disruption of their balance directs cells from survival to apoptotic cell death or from inflammatory to anti-inflammatory responses. Different mechanisms are proposed to explain this yin-yang relationship. Considering the redox aspect of STATs proteins, this review attempts to summarize the current knowledge of redox regulation of STAT1 and STAT3 signaling focusing the attention on the post-translational modifications that affect their activity.

## 1. Introduction

Signal Transducer and Activator of Transcription 1 and 3 (STAT1 and STAT3) are members of a family of seven transcriptional factors (STAT1, 2, 3, 4, 5a, 5b and 6) essential for the cellular response to cytokines and growth factors. They regulate several biological events such as embryonic development, organogenesis, innate and adaptive immunity, cells growth and differentiation, and programmed cell death. In resting cells, STATs proteins are latent in cytoplasm and they translocate to the nucleus after specific stimuli. The classical activation pathway involves the binding of cytokines or growth factors to a specific receptor on cell surface and successive phosphorylation on conserved tyrosine and serine residues by Janus tyrosine kinases (JAKs) and Mitogen-Activated Protein Kinases (MAPKs), respectively. This event allows the dimerization of phosphorylated STATs and their translocation into the nucleus where they modulate the expression of target genes.

The cross-talk between signaling pathways determines how cells integrate the environmental signals received, ultimately translating them in transcriptional regulation of specific sets of genes. STATs transcription factors, being able to detect a variety of environmental signals and to transduce them to the nucleus directly affecting gene regulation, are ideally suited to play a central role in orchestrating the outcome of this cross-talk. Despite being activated downstream of common cytokine and growth factor receptors, STAT1 and STAT3 pathway is reciprocally regulated and perturbation in their balanced expression or phosphorylation levels may redirect cytokine/growth factor signals from proliferative to apoptotic, or from inflammatory to anti-inflammatory.

This review attempts to summarize the current knowledge of redox regulation of STAT1 and STAT3 signaling focusing the attention on the post-translational modifications that affect their activity.

## 2. Signal Transducer and Activator of Transcription 1 and 3

### 2.1. STAT1 and STAT3 Structure

STAT1 and STAT3 proteins are composed of 750 and 770 amino acids, respectively. The 3D-structure of both proteins has been resolved and two crystals for each one are deposited on the Protein Data Bank: the unphosphorylated STAT1 (1YVL) and the Tyr701-phosphorylated STAT1-DNA complex (1BF5) and the unphosphorylated STAT3 core fragment (3CWG) and the phosphorylated STAT3-DNA complex (1BG1).

**Figure 1:**
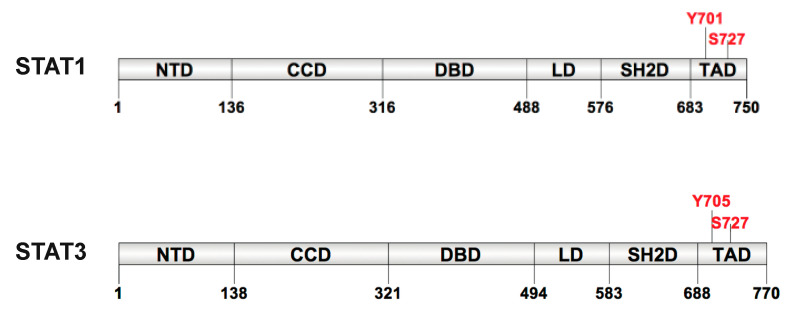
Schematic representation of STAT1 and STAT3 domains. STATs structure is composed by N-domain (NTD), coiled-coil domain (CCD), DNA binding domain (DBD), linker domain (LD), Src homology 2 domain (SH2D) and transcriptional activation domain (TAD). Between SH2D and TAD there is a tail segment that contains the Tyrosine and Serine phosphorylation sites.

The STATs proteins have the same structural and functional domain organization constituted by six functional domains: an N-terminal domain (NTD), a coiled-coil domain (CCD), a DNA-binding domain (DBD), a α-helical linker domain (LD) a Src homology 2 domain (SH2D) and a C-terminal transcriptional activation domain (TAD) (Figure 1).

**Figure 2:**
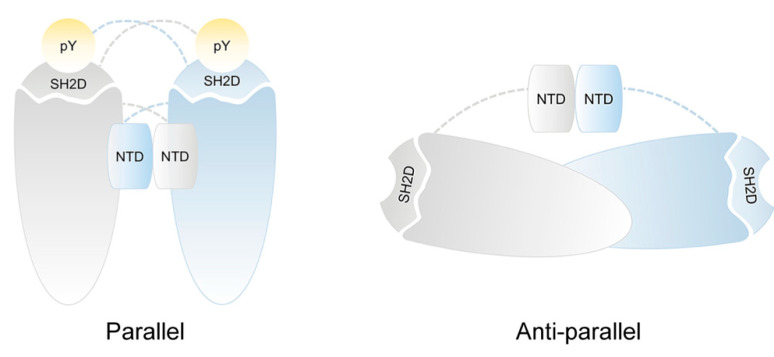
Schematic representation of STAT1 and STAT3 dimeric structure. The cartoon illustrates the proposed dimer conformations for STAT1 and STAT3 dimer: parallel and anti-parallel form. Phosphorylated STAT1 and STAT3 dimerize in parallel form by reciprocal interactions between phosphotyrosine residues and SH2D domains. Non-phosphorylated STAT1 and STAT3 dimers have an anti-parallel orientation of the bodies with SH2D domains on the opposite end of the dimers and the NTD domains lodged between monomer.

They are unique among transcription factors in containing a phosphotyrosine binding SH2D that is the most conserved among STAT family due to its key role in receptor recruitment, phosphorylation and dimerization. The differences in SH2D define the selective receptor binding on each STATs. The SH2D consists in an antiparallel β-sheet with two α-helixes alongside forming a pocket containing a crucial arginine residue (Arg602 in STAT1 and Arg609 in STAT3) that mediates the interaction with the phosphate group [1]. These structural features make the SH2D able to recognize the phospho-tyrosine residues on specific protein regions determining the association with the activated JAKs and the reciprocal SH2-phosphotyrosine interactions of monomeric STATs to form homo- or heterodimers. This conformation is now referred to as a “parallel” STATs dimer. It is important to note that the presence of tyrosine phosphorylation stabilizes this dimer conformation while the absence of phosphate group requires a new rearrangement of the dimer [2,3]. Indeed, the study of unphosphorylated STAT1 crystal structure (PDB:1YVL) reveals a homodimer with a distinct head-to-head orientation of each monomer forming a different dimer structure called “anti-parallel” with the SH2 domains of each monomer at opposite extremities of the dimer (Figure 2) [4,5]. Moreover, the analysis of the crystal structure of the unphosphorylated STAT3 (PDB:3CWG) shows similarity to the antiparallel boat-like structure of STAT1 [6]. Other than SH2D interaction, we have recently detected two interchain disulfides between cysteine 367 and cysteine 542 and between cysteine 418 and cysteine 426 (Cys367-Cys542 and Cys418-Cys426) responsible for STAT3 dimer stabilization [7].

Another highly conserved motif is the NTD, involved in the active dimer formation, in the interaction with transcription co-activators, in the regulation of nuclear translocation and in cooperative binding to DNA. The CCD, formed by a four-helix bundle, is critical for recruitment of STAT1/3 to the receptor and subsequent phosphorylation as well as dimerization, and contains residues essential for nuclear translocation and interaction with regulatory proteins.

Residues 300 to 500 comprise the DBD, a region of beta-sheet structures that binds a specific DNA sequence, called Gamma-interferon Activated Site (GAS). Although contact with DNA is limited, it occurs in both major and minor groove. The LD, originally described as an SH3 homology region, consists of a highly helical fold and constitutes one of the most conserved regions in the STAT molecules [8]. It has been described that mutation in LD affects the DNA binding and switch off signaling after INF-γ stimulation [9]. The TAD, the least conserved domain among the STATs, can be absent due to alternative splicing, contributes to functional specificity and transcriptional activation of target genes [2,10,11,12,13,14]. It is involved in transcriptional activation and promotes the activation of STAT1 and STAT3 through the phosphorylation of conserved Tyr residue (Tyr701 in STAT1 and Tyr705 in STAT3) that controls dimerization by intermolecular interaction with the SH2 domain of the dimer partner. In addition, TAD contains a second conserved phospho-amino acid residue at the C-terminus, a phospho-Ser (Ser727), that is essential for maximal transcriptional activation of STAT.

### 2.2. STAT1 and STAT3 Signaling Cascade

STATs proteins are latent in the cytoplasm of resting cells, either as monomers or unphosphorylated dimers until they are activated by growth factors and cytokines that bind to a specific cell receptor. STATs 1 and 3 signaling can be induced by either distinct or by the same set of cytokines and growth factors (Table 1).

Despite being activated downstream of common cytokine and growth factor receptors, STAT1 and 3 activation is often cell type and ligand specific and, in a lot of conditions, play opposing roles in regulating cancer cell growth, proliferation, cell survival, apoptosis and immune response. Each STAT mediates its biological effects by trans-activating a unique profile of target genes dependently on the interactions with STAT-associated regulatory factors [15,16,17].

**Figure 3:**
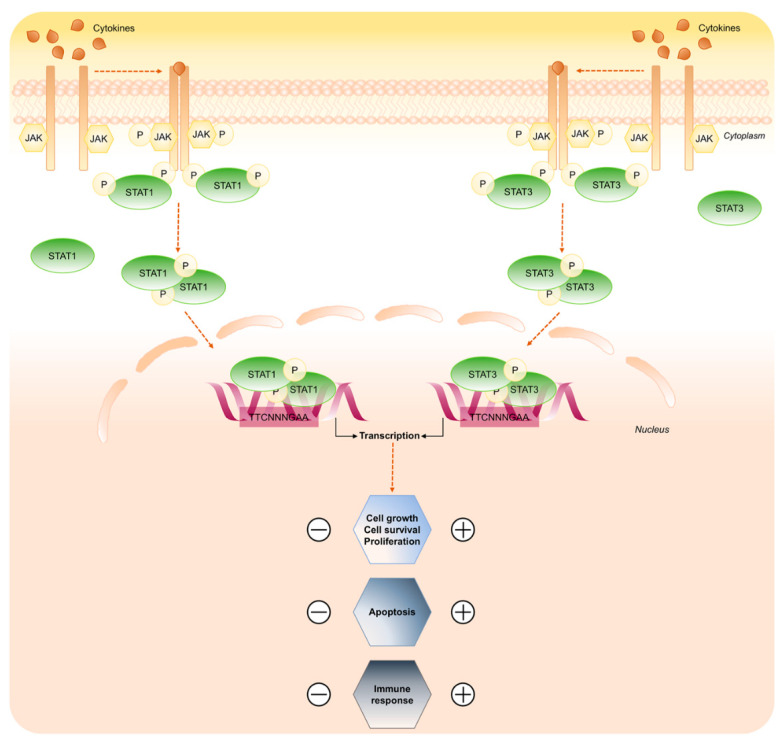
STAT1 and STAT3 signaling pathway. Key steps of STAT1 and STAT3 signaling in response to cytokines. Binding of the ligands to the receptors induces receptor dimerization and activation of receptor associated JAK kinase, which in turn phosphorylates STAT1 and STAT3 proteins. Active STAT1/3 form dimer and translocate to the nucleus to control gene expression. The scheme focuses on the opposing roles of STAT1 and STAT3 transduction cascade.

The classical pathway of signal transduction mediated by STATs factors is activated through the binding of the ligands to their cognate cell-surface receptors. This allows the rapid trans-phosphorylation and activation of the JAKs kinases (JAK1, JAK2, JAK3 and TYK2) constitutively associated with the cytoplasmic membrane-proximal regions of various receptors. The receptor phospho-tyrosine residues manage the SH2-dependent recruitment of specific STATs, which in turn become JAKs substrates. STATs are phosphorylated on conserved tyrosine residue near the C-terminus (Tyr701 in STAT1 and Tyr705 in STAT3) and, after release from the receptor, homo- or hetero-dimerize and enter the nucleus by a mechanism that is dependent on importin-Ran-mediated active transport (Figure 3) [18,19,20].

Although STATs activation by JAKs is the best characterized pathway, STAT1 and STAT3 can also be activated by receptors with intrinsic Tyrosine Kinase (RTK) activity such as EGFR and PDGFR, and by non-receptor tyrosine kinases (NRTKs) other than JAKs, such as Src kinase and ABL [21].

Once in the nucleus, dimerized STAT1 and STAT3 bind specific regulatory sequences, small DNA palindromic consensus sequence (TTCN_2–4_GAA) that marks a GAS element and activate or repress transcription of target genes.

It has been reported that the maximal activation of STAT1 and 3 is achieved when also Ser727 is phosphorylated. On the basis of the cellular setting, stimulus, and STAT member, several serine kinases, including ERK1/2, p38 and pKC-d, are involved. Yet, adverse findings ascribe to Ser phosphorylation a negative regulation on DNA binding [22].

This apparently linear order of events is indeed made complex by a lot of signals integration from multiple cytokine receptors, mutations, selective dimerization, negative pathway regulation, and post-translational modification of pathway members.

### 2.3. Negative Regulation of STAT1 and STAT3 Signaling

**Figure 4:**
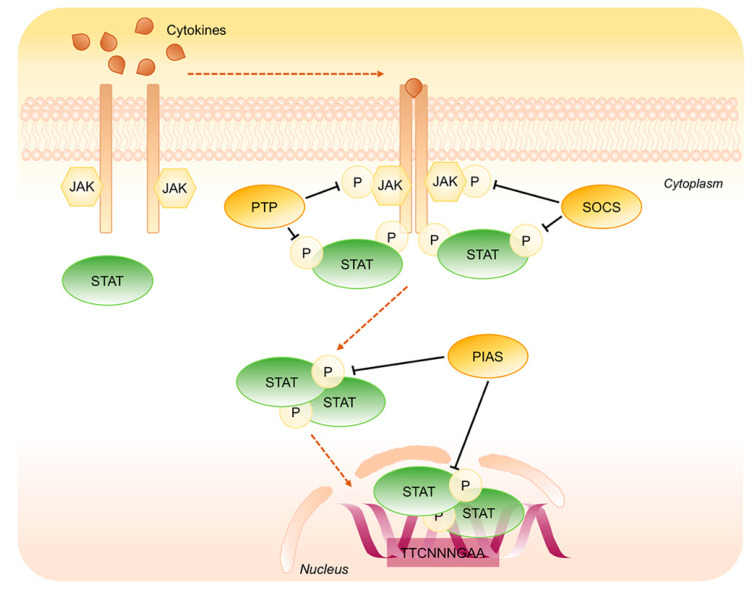
Negative regulation of STAT1 and STAT3 signaling pathway. Key steps of STAT1/3 signaling negative regulation. Phosphotyrosine Phosphatases (PTP) and Suppressors of Cytokine Signaling (SOCS) dephosphorylate JAKs as well as STATs in the cytoplasm whereas the Inhibitory Proteins of Activated STATs (PIAS) operate on STATs dimers in the cytoplasm as well as into the nucleus.

A number of studies has identified negative regulatory mechanisms that exist to curtail the activity of STAT proteins. These include the action of phosphotyrosine phosphatases (PTP), suppressors of cytokine signaling (SOCS), interaction with inhibitory proteins such as protein inhibitor of activated STATs (PIAS), and targeted proteasome-dependent degradation of active STATs (Figure 4).

#### 2.3.1. Phosphotyrosine Phosphatases

The PTP family is involved in the dephosphorylation of JAKs and STATs proteins. Their function is of crucial importance to fine-tune the activities of STATs factors, but only some PTP-dependent modulation of STAT signaling have been studied thoroughly. The best characterized of these proteins are SHP1 and SHP2, featuring SH2 domain that recognizes the phosphotyrosine residues. Among them, SHP1, mainly expressed in the nucleus of epithelial cells and in the cytoplasm of hematopoietic cells, is responsible for JAKs and STATs inactivation and SHP2, ubiquitous and present both in the cytoplasm and in the nucleus, is able to inactivate both STAT1 and STAT3. SHP2 can directly inactivate STAT1 dephosphorylating both p-Tyr701 and p-Ser727 residues. Indeed, the knockout of SHP2 in mouse fibroblast cells outcomes in enhanced and prolonged STAT1 phosphorylation at both Tyr701 and Ser727 [23]. There is also evidence that SHP2 negatively regulates the activity of STAT3 since SHP2^Δ/Δ^ mutant mouse ES cells, in the presence of leukemia inhibitory factor (LIF), showed defective differentiation and more efficient self-renewal, partly because of an increased in STAT3 activity in the absence of functional SHP2 [24]. Others PTP involved in the inhibition of STAT1 signaling are PTP1B, a specific inhibitor of JAK2 and TYK2 and T-Cell PTP (TCPTP) that acts on JAK1. The nuclear isoform of TCPTP, called TC45, not only directly dephosphorylates p-Tyr701-STAT1 inducing its export from the nucleus but also regulates IL6-mediated signaling pathway through STAT3 dephosphorylation [25,26]. Interestingly, the tumor suppressor activity of two further PTPs has recently been linked to STAT3 inactivation: PTPRD was found frequently inactivated and unable to dephosphorylate the oncoprotein STAT3 in glioblastoma and in other human cancers and PTPRT was shown to reduce IL-6 mediated STAT3 activation in HCT116 cell lines [27,28].

#### 2.3.2. Suppressors of Cytokine Signaling

The SOCS family includes eight intracellular proteins that have a key role in protein degradation process through the ubiquitin/proteasome pathway. SOCS proteins, such as SOCS1 and SOCS3, are characterized by the presence of a central SH2 domain and by a conserved region, called SOCS BOX, that directly binds the ubiquitin E3 ligase complex leading to kinases degradation [29]. The SOCS expression is quickly induced by the same cytokines that trigger the JAK/STAT pathway causing the inhibition of STATs signaling through a negative feed-back mechanism. Among the SOCS family, SOCS1 and SOCS3 are unique in that they contain a 12 amino acid region known as the kinase inhibitory region (KIR), required for inhibition of JAK kinase activity [30,31]. SOCS1 is the most potent member of the SOCS family and it is shown to be a direct inhibitor of the catalytic activity of JAK1, JAK2 and TYK2 but not JAK3. Another member of the same protein family, the Cytokine-Induced SH2 domain protein (CIS), competes with STATs for the binding to the tyrosine residues within the receptor cytoplasmic domains [32,33].

#### 2.3.3. Protein Inhibitor of Activated STATs

The PIAS family consists of four members (PIAS1, PIAS3, PIASX and PIASY) that interact with STATs activated dimers preventing their association with DNA and gene transcription [34]. The specific PIAS-STAT interaction is cytokine-dependent and, within the PIAS family, the different proteins act through different mechanisms. PIAS1 and PIAS3 were identified as interaction partners for STAT1 and STAT3, respectively and have been shown to function also through a SUMO E3 ligase-like mechanism promoting the conjugation of transcriptional factors with the Small Ubiquitin-like Modifier (SUMO) [35]. Otherwise PIASY is a transcriptional corepressor of STAT1 and its LXXLL coregulator signature motif, located at the NH2 terminus, is involved in the modulation of STAT-mediated gene expression [36].

### 2.4. Post-Translational Modification

Aside from the phosphorylation, STATs activity can be regulated by other post-translational modifications such as ubiquitination, acetylation, methylation, S-glutathionylation and ISGylation.

#### 2.4.1. Ubiquitination

Protein ubiquitination plays a role in controlling cellular signaling pathways by marking proteins, including transcription factors, for degradation through the 26S proteosome-dependent pathway. A STAT-interacting LIM protein (SLIM) was identified, as a STAT ubiquitin E3 ligase, for regulating the STATs signaling pathway [37]. It has been suggested that SLIM/PDLIM2 E3 ligase targets STAT3 [38]. Yuan et al. provided evidence that Smurf1 targets STAT1 for ubiquitination and proteasomal degradation with a negative feedback regulation on IFNγ signaling [39]. In prostate cancers STAT3 was identified as a substrate of E3 ubiquitin ligase COP1, which is involved in its ubiquitination and degradation [40]. Otherwise, Ray et al. demonstrated that the mono-ubiquitination of Lys94 residue in the NH2-terminal domain of STAT3 augments its transcriptional activity [41].

#### 2.4.2. Acetylation

Histone acetyltransferase CBP prompts the acetylation of Lys410 and Lys413 residues of STAT1 leading to dissociation from the promoter sequences and induces binding of phosphatase TCP45, which catalyzes dephosphorylation [42]. Conversely, the STAT3 acetylation of Lys49 and Lys87 located at NH2 terminal and of Lys685 at -COOH terminal is associated with positive regulation and DNA binding [43].

#### 2.4.3. Methylation

Mowen et al. reported that STAT1 methylation on Arg31, mediated by Protein Methyl-Transferase 1 (PRMT1), is functionally necessary for full activation of transcription in response to IFNα/β receptor since it suppresses the interaction with the negative regulatory protein PIAS1 [44]. Moreover, Chen et al. identified another site of methylation on Lys525 catalyzed by the methyltransferase STD2 as an essential signaling event for IFNα-dependent antiviral immunity. Nevertheless, STAT1 methylation needs to be explored more thoroughly because it is at the moment controversial [45].

STAT3 is reversibly methylated on Lys residues by histone-modifying enzymes, with important functional consequences [46]. It is known to be di- or trimethylated on Lys140 or Lys49 and Lys180 by the histone methyltransferase SET9 (SET domain containing lysine methyltransferase 9) or EZH2 (enhancer of zeste homolog 2), respectively [47]. Methylation on Lys140 blocks the binding of STAT3 to DNA, negatively affecting STAT3-dependent transcription [47]. Otherwise, Lys49 and Lys180 methylation enhances STAT3 tyrosine phosphorylation by protecting it from dephosphorylation, positively affecting STAT3-dependent transcription [48].

#### 2.4.4. S-Glutathionylation

STAT1 and STAT3 are both gluthationylated on specific cysteine residues in response to a perturbation in the intracellular redox environment [49,50,51]. Intriguingly, this post translational modification exerts opposite roles on the transduction cascade of both transcription factors. This will be discussed in detail below.

#### 2.4.5. ISGylation

Protein ISGylation is a ubiquitination-like post-translational modification that covalently conjugates Interferon-Stimulated Genes (ISG) peptides to lysine residues of target proteins. ISG15 is highly induced by type I interferons and it is involved in the positive regulation of JAK/STAT1 pathway. It is unclear so far, whether other STAT family members involved in IFN signaling, such as STAT3, are also subjected to ISG15 conjugation [52].

## 3. Redox Regulation of STAT1 and STAT3 Signaling Cascade

### 3.1. Oxidative Post-Translational Modifications of Proteins

Over the last two decades, oxidative post-translational modifications of proteins have emerging as an important signaling mechanism that regulates different cellular functions in physiological as well as pathological conditions and aging [53]. The consequences of protein oxidation depend on the type and concentration of oxidants involved and vary from protein to protein depending on their specific biochemical and structural characteristics [54]. Indeed, protein oxidation modifies size, hydrophobicity, charge or polarity of specific amino acids in protein affecting its secondary and tertiary structure and dictating the stability and activity of the whole protein.

**Figure 5:**
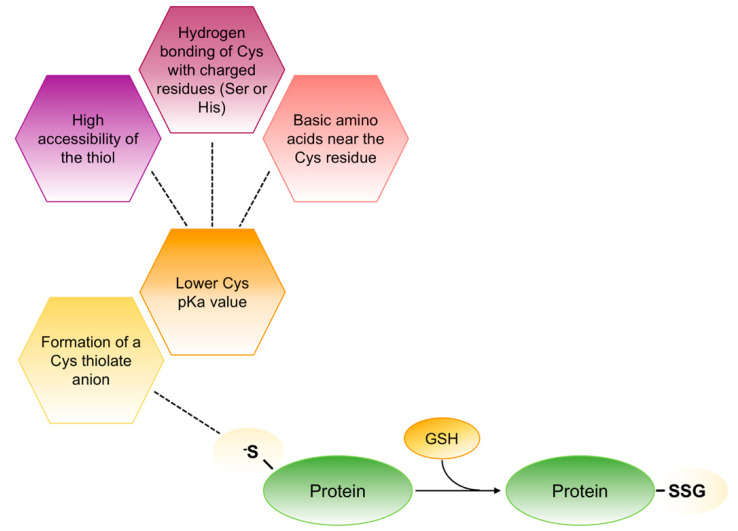
Schematic representation of thiolate anions formation in redox-sensitive proteins. In redox-sensitive proteins different factors contribute to lower the pK_a_ value associated to the thiol side chain of cysteine that forms the thiolate anions (S^−^). This anion is stable as such to react with GSH to form mixed disulphides.

Due to their related redox properties, cysteine residues appear to be the principal target sites for oxidants. Specifically, at physiological pH the thiolic groups of one or more cysteines in redox-sensitive proteins exist as reactive thiolate anions that are more nucleophilic and more susceptible to oxidation that the non-ionized form. Different factors are involved in this process but all of them contribute to the decrease of the pK_a_ value associated to the thiol side chain of cysteine that usually is ∼8.25 (Figure 5). Therefore, the reactive thiol of cysteine may function as a sensor or switch, that flips between the reduced and oxidized state in response to altered localized redox potential [55].

**Figure 6:**
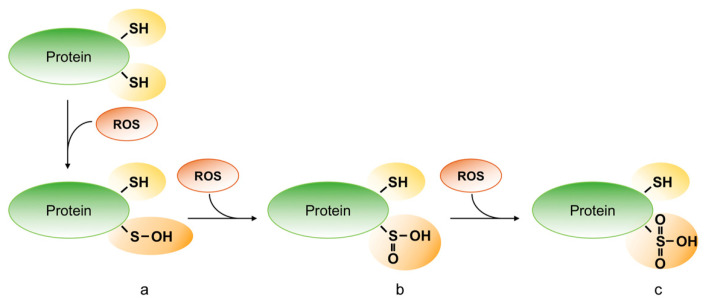
Oxidative modifications of protein thiols. Reactive oxygen species (ROS) can oxidize the protein on the thiol group of cysteine residues and can generate sulfenic (R-SOH) (**a**), sulfinic (R-SO_2_H) (**b**) and sulfonic acid (R-SO_3_H) (**c**).

Different oxidative post-translation modification of cysteine may occur in cells depending on types, amounts and cellular localization of oxidants. Thus, reactive oxygen free radical (e.g., superoxide, O^−2^; hydroxyl, ·OH; peroxides ROO) and non-radical (e.g., hydrogen peroxide) species (ROS) oxidize the thiol of cysteine and generate sulfenic (R-SOH), sulfinic (R-SO_2_H) and sulfonic acid (R-SO_3_H) (Figure 6).

**Figure 7:**
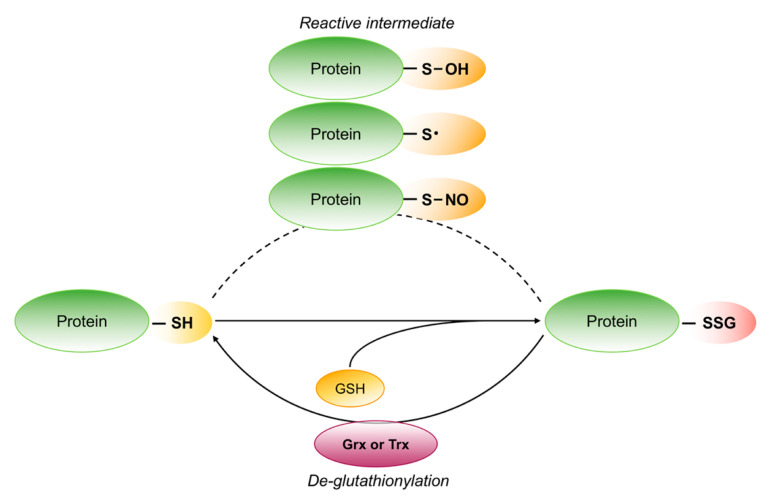
Schematic representation of reversible protein S-glutathionylation. The thiol groups on reactive cysteine are S-glutathionylated after the formation of different reactive intermediate. S-glutathionylation can be reversed by glutaredoxin (Grx) or thioredoxin (Trx).

These different oxidation states of proteins may result in diverse biological outcome causing the fate of cells [56]. Indeed, RSOH may rapidly react with nearby thiol of the same protein or with other thiol to form intra-or intermolecular disulfide bonds. Specifically, when RSOH react with GSH, one of the most abundant low molecular mass thiols in cells, the result is S-glutathionylation, a reversible post-translational modification of protein. This modification can also occur between the thyil radicals (RS), formed by one electron oxidation of protein or GSH thiol group, and the -SH group of GSH or protein. S-glutathionylation can be reduced back by glutaredoxins (Grx) or thioredoxins (Trx) (Figure 7) [57,58,59,60,61]. These reversible processes permit redox signaling and may protect proteins from irreversible oxidation.

On the other end, R-SO_2_H and R-SO_3_H, that result from further oxidation, may cause an irreversible damage of proteins with permanent alteration of their function.

The thiol groups can also be oxidized by reactive nitrogen species (RNS) to form reversible S-nitrosothiols (R-SNO), characterized by a short half-life as a consequence of spontaneous de-nitrosylation by excess GSH that can generate GSNO or glutathionylated protein (Figure 7). However, few S-nitrosylated proteins become stable since S-nitrosylation induces conformational changes that limit GSH accessibility [62].

### 3.2. Redox Regulation of STAT1

Many reports suggest that STAT1 is a redox-sensitive protein, but the biological outcomes vary according to oxidative insults and biological system examined. It has been reported that oxidative stress caused by H_2_O_2_ treatment inhibits STAT1 phosphorylation in neuronal cells but had no effect in non-neuronal ones [63]. On the other hand, H_2_O_2_ and/or derived ROS directly activate STAT1 in cultured astrocytes [64], glia [65] and in vascular smooth muscle cells where STAT1 signaling promotes cell growth [66].

Furthermore, Terui et al. describe that hypoxia and reperfusion treatment induces redox-dependent STAT1 phosphorylation in primary rat hepatocytes and demonstrate that STAT1 activation is involved in cell survival [67]. Conversely, it has been described that STAT1 activation is critical involved in ischemic cardiomyocytes cells apoptosis [68]. The redox dependent activation of STAT1 is also described in outer membranes of basilar arteries in animal model of subarachnoid hemorrhage [69] and in cardiomyocytes in mouse myocardial ischemia/reperfusion injury model [68]. The ability of H_2_O_2_ to activate STAT1 cascade has been associated to an increase of JAK2 and TYK2 tyrosine kinase activity [70,71,72]. Although many authors associate the ability of oxidative stress to activate STAT1 cascade to an increase of JAK2 and TYK2 tyrosine kinase activity, Grohmann et al. demonstrate that obesity-induced oxidative stress inhibits the protein tyrosine phosphatase activity resulting in activation of STAT1 [73]. Therefore, all most relevant literature evidences point out the role of tyrosine kinase or phosphatase in redox regulation of STAT1 signaling without focusing the attention on STAT1 protein itself.

We recently demonstrated that H_2_O_2_ or hypoxia treatment rapidly induces tyrosine phosphorylation and cysteine glutathionylation of STAT1 in mouse microglia BV2 cells [51,74]. The analysis of the molecular mechanism of STAT1 signaling revealed that both phosphorylation and glutathionylation contribute to activation of STAT1 during oxidative stress. Mass mapping investigations indicated Cys324, in the DBD domain, and Cys492, between the DBD and LD domains of STAT1 structure, as the main targets of S-glutathionylation in recombinant purified STAT1. It is important to observe that the addition of the bulky negatively charged GSH moieties in the DBD domain does not hamper JAK2 mediated STAT1 tyrosine phosphorylation and STAT1 DNA binding ability. A deep biochemical cellular study of the molecular mechanism of STAT1 redox regulation confirms that Cys324 and Cys492 are the main targets of S-glutathionylation [51]. Our data propose S-glutathionylation of STAT1 as a novel mechanism by which oxidative stress regulates STAT1 signaling in microglia cells. This molecular mechanism has to be confirmed in another cellular model.

### 3.3. Redox Regulation of STAT3

STAT3 is a redox-regulated protein and its function is strictly affected by intracellular redox environment. Although many reports confirm the influence of oxidative stress in STAT3 cascade, it is still not clear if ROS up- or down-regulate STAT3 activation. Some authors report an increase of Tyr705 phosphorylation of STAT3 and up-regulation of its DNA-binding activity after different oxidative stimuli [75,76]. In addition, the treatment of cells with ROS scavengers or NADPH oxidase enzyme inhibitors, is able to prevents STAT3 activation [77]. On the other hand, many reports demonstrate that transcriptional activity of STAT3 is impaired by oxidative stress through the oxidation of conserved cysteine residues in STAT3 DBD domain [78,79]. Moreover, Zgheib et al. show that the activity of STAT3 is inhibited by nitrosocyclohexyl acetate, a nitroxyl donor, through the formation of sulfenic acid at the cysteine residues in endothelial cells [80].

Two important redox post-translational modifications in STAT3 protein, S-glutathionylation and S-nitrosylation, have been found to inhibit its signaling pathway. They are able to hinder STAT3 phosphorylation as well as its DNA binding activity in different cell lines and in in vitro model.

The S-glutathionylation of STAT3 was firstly described in 2009 by Xie et al. The authors revealed that this post-translational modification inhibits the phosphorylation of transcription factor and impairs expression of its target genes in human HepG2 hepatoma cells [50]. Subsequently, our group identified three sesquiterpene lactones able to down-modulate the activation of STAT3 through the disruption of intracellular redox homeostasis and induction of S-glutathionylation of STAT3 [81,82,83]. Deepening inside the redox regulation of STAT3 signaling, in 2014, we identified the Cys328 in the DBD domain, and Cys542 in the LD domain as target of S-glutathionylation [49]. Although the crystal structure of glutathionylated STAT3 is not available, it can be speculated that the addiction of GSH on STAT3 could induce a slight modification in its structure hindering accessibility to JAK2 and consequently its phosphorylation [49,50,51,79].

The S-nitrosylation of STAT3 was discovered in 2015 by Kim et al. [79]. The authors demonstrated that the addiction of NO to cysteine residues down-modulates STAT3 signaling pathway. Furthermore, they identified Cys259 in the CCD domain of STAT3 as target of S-nitrosylation and suggested that NO adduct perturbs domain folding impairing interaction between STAT3 and its receptor (phospho-Tyr of gp130) and successive JAK2-mediated tyrosine phosphorylation of STAT3.

Redox regulation of STAT3 also occurs via oxidation and reduction of exposed Cys residues, through a redox relay involving peroxiredoxin-2 (Prx2) and thioredoxin-1 (Trx1) [83,84]. Following induction of oxidative stress by H_2_O_2_, Prx2 rapidly oxidizes STAT3 inducing the formation of unphosphorylated STAT3 dimer transcriptionally inactive. The restore of STAT3 transcriptional activity is due to Trx1 action that reduce oxidized STAT3 dimers and re-establishes the phosphorylation state of STAT3 [83].

## 4. Concluding Remarks

This review aims to provide an overview on the oxidation mechanism of cysteine residues in the proteins and to discuss the redox regulation of STAT1 and STAT3 signaling.

It is well known that STAT1 and STAT3 play essentially antagonistic roles and the disruption of their balance directs cells from survival to apoptotic cell death or from inflammatory to anti-inflammatory responses. Many authors describe a conflicting function of STAT1 and STAT3 in ischemic heart disease where STAT1 contributes to the loss of cardiomyocytes and STAT3 protects them from I/R injury [68,85,86,87,88]. Analogously, in neurodegenerative disorders STAT1 exerts a pro-apoptotic effect contributing to brain damage whereas STAT3 shows neuroprotective properties through the inhibition of apoptosis [89,90].

**Figure 8:**
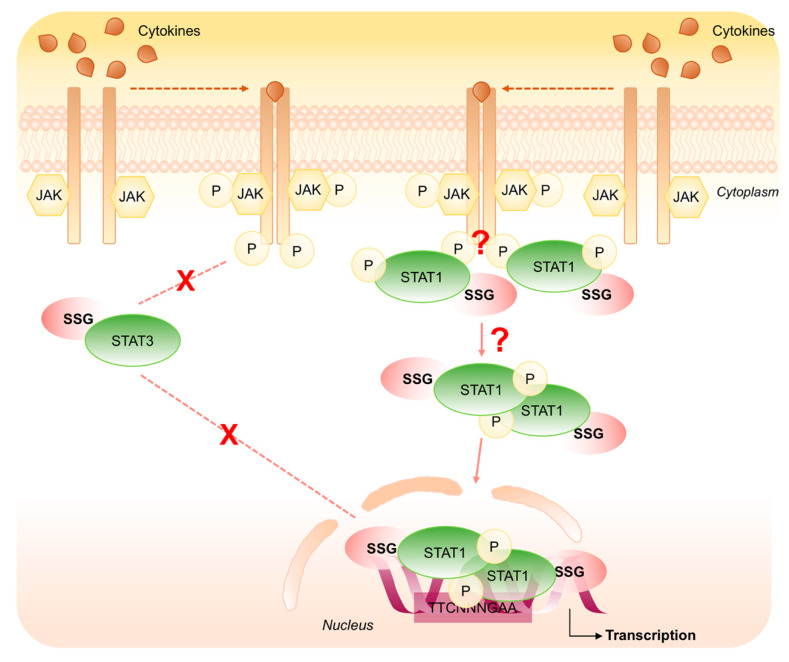
Schematic representation of the opposing role of S-glutathionylation on STAT1 and STAT3 signaling cascade. Oxidative stress induces STAT3 S-glutathionylation impairing its phosphorylation and down-regulating its signaling (dashed line). Conversely, oxidative stress induces both phosphorylation and S-glutathionylation of STAT1 that are responsible of its aberrant activation (solid line).

Different mechanisms are proposed to explain this yin-yang relationship between STAT1 and STAT3 signal transduction cascade. Considering the redox aspect of STATs proteins, in this review we highlight the importance of S-glutathionylation that affects phosphorylation and dephosphorylation of STAT1 and STAT3 exerting an opposing role in the modulation of the transduction pathway. Specifically, modification in the intracellular redox environment induces STAT3 S-glutathionylation impairing its phosphorylation and down-regulating its signaling. Conversely, oxidative stress induces both phosphorylation and S-glutathionylation of STAT1 that are responsible of its aberrant activation (Figure 8).

Taking into account the opposing role of STAT1 and STAT3 signaling in the development of different cardiovascular diseases as well as in neurodegenerative disorders, therapeutic strategies that target STAT1/3 S-glutathionylation may offer new promise for drug development also in combination with more standardized therapies.

## Figures and Tables

**Table 1:** Cytokines, growth factors and related JAKs involved in the activation of STAT1 and STAT3 signaling.

Cytokines	JAKs	STATs
IFN-g	JAK1, JAK2	STAT1
NP	JAK1, (JAK2)	STAT3
CT-1	JAK1, (JAK2)	STAT3
G-CSF	JAK1, (JAK2)	STAT3
Leptin	JAK2	STAT3
GH	JAK2	STAT3
IFN-a/b	JAK1, TYK2	STAT1/STAT3
IL-6	JAK1, (JAK2)	STAT1/STAT3
IL-9	JAK1, JAK3	STAT1/STAT3
IL-10	JAK1, TYK2	STAT1/STAT3
IL-11	JAK1	STAT1/STAT3
IL-12	TYK2, JAK2	STAT1/STAT3
IL-19	JAK1, JAK2	STAT1/STAT3
IL-20	JAK1, JAK2	STAT1/STAT3
IL-21	JAK1, JAK3	STAT1/STAT3
IL-22	JAK1, TYK2	STAT1/STAT3
LIF	JAK1, (JAK2)	STAT1/STAT3
CNTF	JAK1, (JAK2)	STAT1/STAT3
OSM	JAK1, (JAK2)	STAT1/STAT3

## Abbreviations

CCD	Coiled-Coil Domain
DBD	DNA-binding domain
Grx	Glutaredoxin
GSH	Glutathione
GSSG	Glutathione disulphide
JAK	Janus tyrosine kinases
LD	α-helical linker domain
MAPKs	Mitogen-Activated Protein Kinases
NTD	N-terminal domain
PIAS	Protein Inhibitor of Activated STATs
Prx2	Peroxiredoxin-2
PTP	Phosphotyrosine phosphatases
RNS	Reactive Nitrogen Species
ROS	Reactive Oxygen Species
R-SNO	S-nitrosothiols
R-SOH	Sulfenic acid
R-SO_2_H	Sulfinic acid
R-SO_3_H	Sulfonic acid
SH2D	Src homology 2 domain
SOCS	Suppressors of Cytokine Signaling
STAT1	Signal Transducer and Activator of Transcription 1
STAT3	Signal Transducer and Activator of Transcription 3
TAD	C-terminal transcriptional activation domain
Trx	Thioredoxin
